# Robot-Assisted Nephroureterectomy for Urothelial Carcinoma in a Horseshoe Kidney: A Case Report

**DOI:** 10.7759/cureus.89506

**Published:** 2025-08-06

**Authors:** Takayuki Ohzeki, Yuto Mitsuhashi, Yoshio Ohta, Saizo Fujimoto, Kazutoshi Fujita

**Affiliations:** 1 Department of Urology, Izumi City General Hospital, Izumi, JPN; 2 Department of Diagnostic Pathology, Izumi City General Hospital, Izumi, JPN; 3 Department of Urology, Kindai University, Sayama, JPN

**Keywords:** da vinci xi® system, horseshoe kidney, minimally invasive urologic surgery, renal anomalies, robot-assisted nephroureterectomy, upper tract urothelial carcinoma

## Abstract

The horseshoe kidney is the most common renal fusion anomaly, and its unique anatomical configuration and aberrant vasculature present significant surgical challenges, particularly in malignant conditions such as urothelial carcinoma. We report a case of robot-assisted right nephroureterectomy with the da Vinci Xi® Surgical System (Intuitive Surgical Inc., Sunnyvale, CA) in a patient with right lower ureteral cancer associated with a horseshoe kidney. The procedure was completed robotically using the da Vinci Xi® system in 207 minutes with minimal blood loss. The pathological diagnosis was high-grade urothelial carcinoma (pT2) with negative surgical margins and one positive lymph node near the ureteral tumor (N1). The postoperative course was uneventful, and no recurrence was observed at 24 months. This case demonstrates the safety and efficacy of robot-assisted surgery for upper tract urothelial carcinoma (UTUC) in patients with complex renal anomalies such as a horseshoe kidney.

## Introduction

Horseshoe kidney is a congenital renal fusion anomaly involving the lower poles, occurring in approximately one in 500 individuals. This anomaly is frequently accompanied by multiple aberrant renal arteries and veins, which complicate surgical management. The incidence of urothelial carcinoma is higher in patients with horseshoe kidneys than in the general population [1].

Due to the anatomical complexity of this condition, detailed preoperative vascular imaging is crucial to avoid complications during surgery. While open surgery was traditionally preferred, the advent of laparoscopic and robot-assisted approaches has expanded surgical options. Robot-assisted systems offer enhanced dexterity, tremor reduction, and 3D visualization, enabling safer and more precise surgery in anatomically complex cases [2,3].

However, robot-assisted radical nephroureterectomy (RANU) for horseshoe kidneys is a rare procedure, with only three such cases previously reported [4,5]. This report describes the fourth such case, emphasizing preoperative planning and intraoperative technique using the da Vinci Xi® system (Intuitive Surgical Inc., Sunnyvale, CA). This article has not been previously presented or published in any form, including abstract, poster, or oral presentation.

## Case presentation

A 71-year-old woman with no known comorbidities and a history of uterine fibroid surgery was referred to our hospital following the detection of right renal pelvic dilatation on abdominal ultrasonography. Urinalysis revealed hematuria and concentrated urine, and urine cytology was classified as class III. Blood test results were within normal limits.

**Figure 1:**
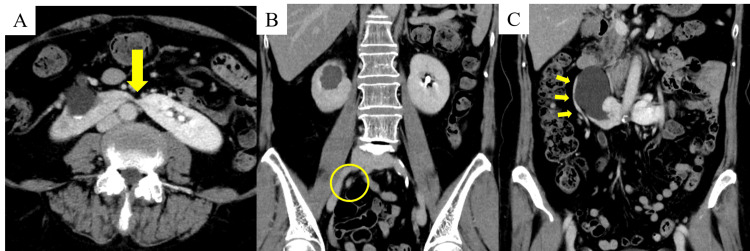
Contrast-enhanced computed tomography (CT) findings (A) Renal hilum of the horseshoe kidney (arrow). (B) Right hydronephrosis and a 5 × 5 mm solid tumor exhibiting contrast enhancement in the right lower ureter. (C) A single vascular branch originating from the distal right renal vein and extending toward the pelvis is observed around the right kidney (arrow).

**Figure 2:**
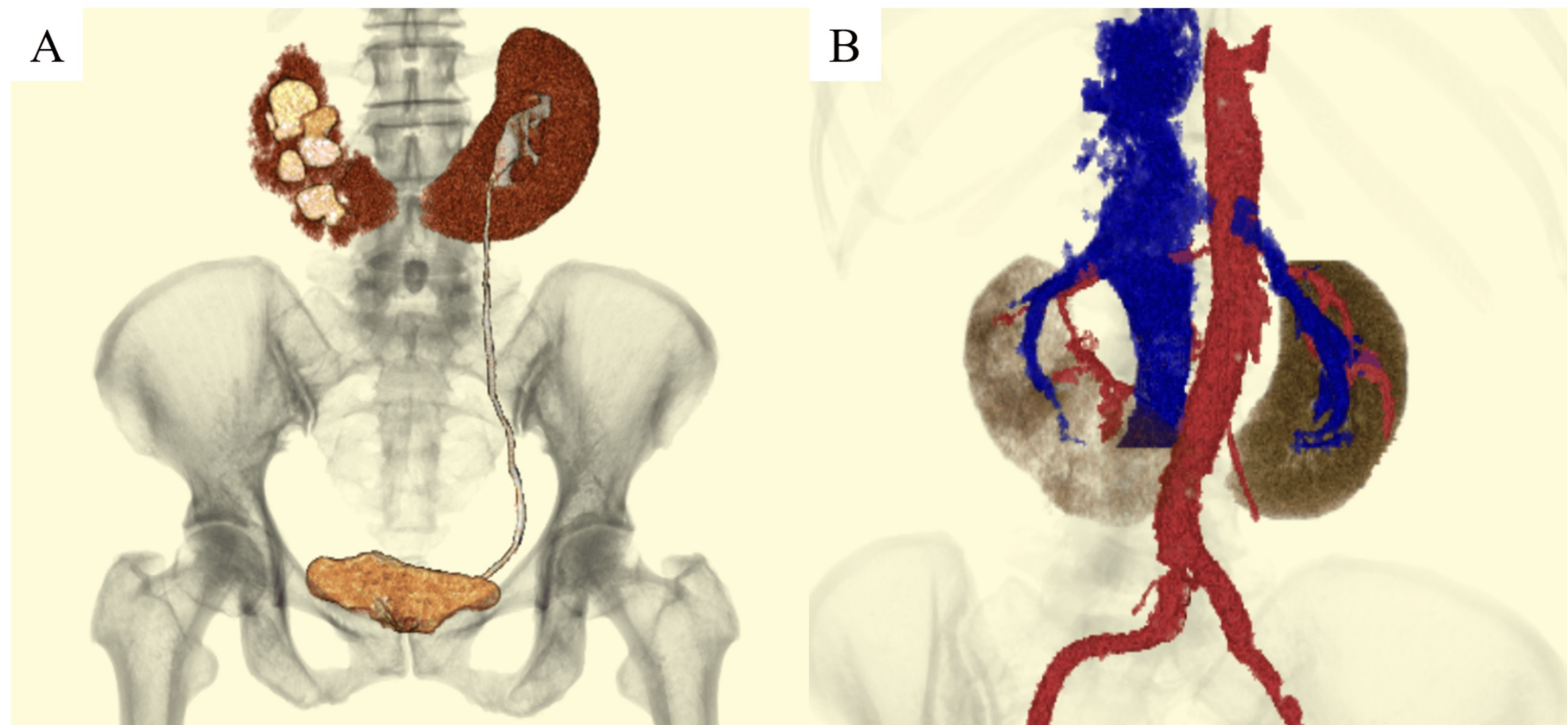
Contrast-enhanced CT three-dimensional (3D) composite image Right hydronephrosis (A) is noted, and the right urinary tract is not visualized. The renal artery and vein are clearly depicted in the vascular composite image (B).

Contrast-enhanced computed tomography (CT) revealed a horseshoe kidney (Figure 1A), mild right-sided hydronephrosis (Figures 1-2A), and a 5 × 5 mm contrast-enhancing solid tumor in the right lower ureter (Figure 1B). No lymphadenopathy or distant metastases were observed. Based on imaging findings, a diagnosis of right lower ureteral cancer with horseshoe kidney (cT2 ≥ cN0M0) was made, and robot-assisted right nephroureterectomy was planned.

Preoperative CT angiography revealed a single right renal artery and vein (Figure 2B), as well as an aberrant vessel branching from the distal right renal vein toward the pelvis (Figure 1C).

**Figure 3:**
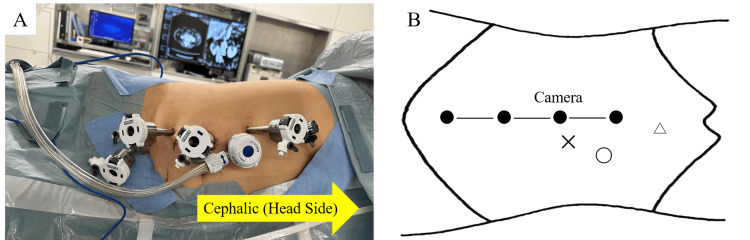
Patient positioning and port configuration during robot-assisted nephroureterectomy (RANU) (A) The patient is positioned in a mild Trendelenburg position. (B) Port layout: ● 8-mm robotic ports (camera and robotic arms) spaced approximately 7 cm apart, 〇 12-mm assistant port (AirSeal™ system), △ 5-mm assistant port. Image credits: Takayuki Ohzeki

**Figure 4:**
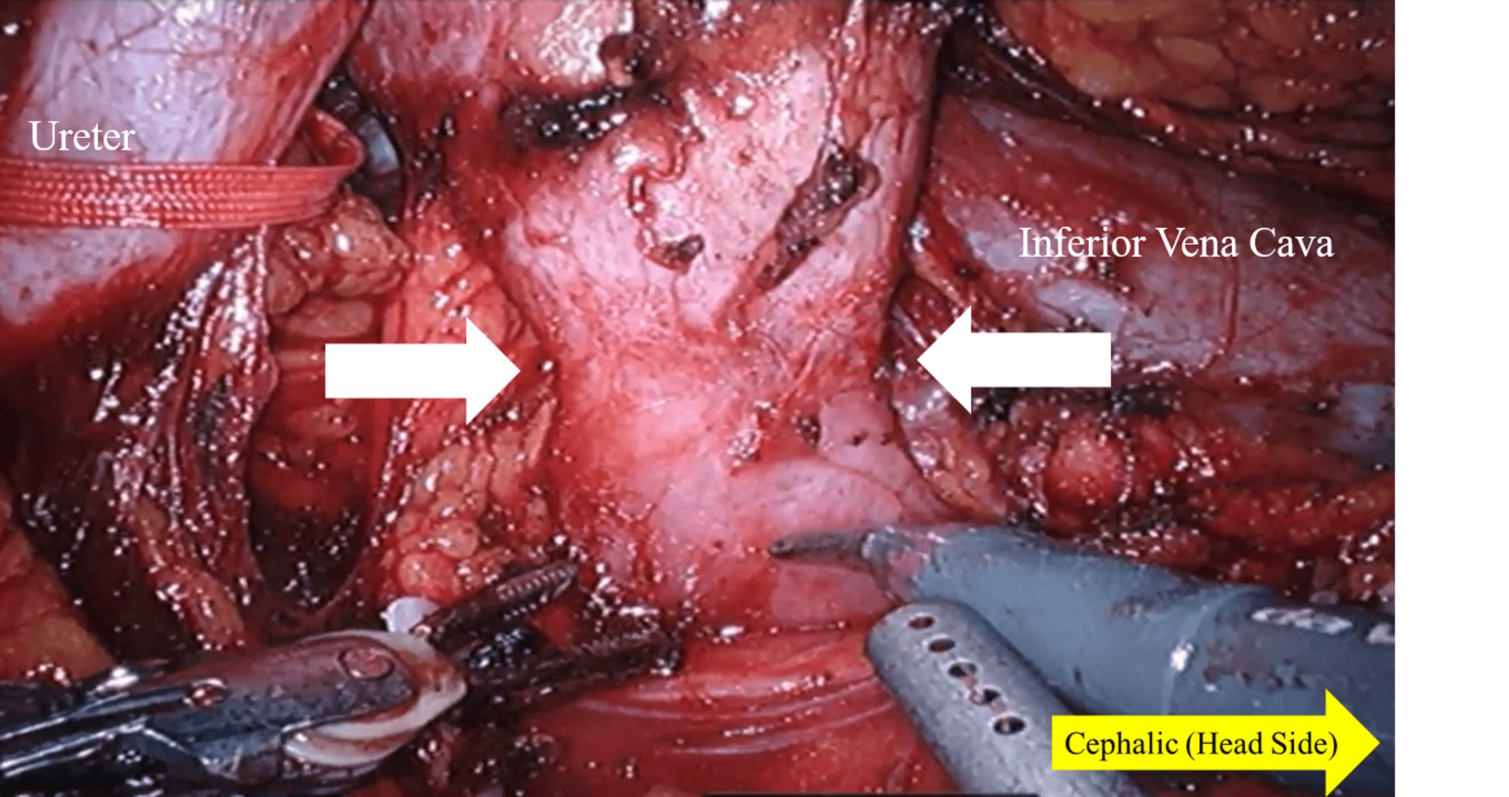
Intraoperative findings in the renal hilum Intraoperative view showing the renal isthmus of a horseshoe kidney. The fused lower poles are visible between the ureter and the inferior vena cava.

The surgery was performed using the da Vinci Xi® robotic system with the patient in the lateral decubitus position. Four da Vinci robotic ports were placed in a parallel configuration, each spaced approximately 7 cm apart. Additionally, two assistant ports were inserted separately. (Figure 3). A transperitoneal approach was utilized. The aberrant vessel was ligated and transected using Hem-o-lok® clips (Teleflex Medical, Research Triangle Park, Durham, NC). The isthmus of the horseshoe kidney (Figure 4) was divided with monopolar coagulation and closed with continuous 3-0 multifilament sutures. The resection was bloodless and performed under clear visualization. Lymph node dissection was not routinely performed in accordance with guidelines, which recommend a selective approach based on risk stratification.

The total operative time was 207 minutes (console time: 150 minutes), and the estimated blood loss was 75 mL. The patient experienced no postoperative complications and was discharged on postoperative day 5.

**Figure 5:**
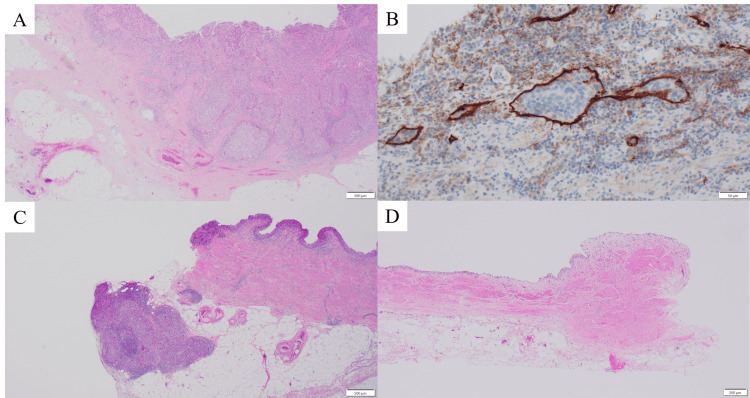
Pathological findings (A) Hematoxylin and eosin staining (×20): Invasion of urothelial carcinoma into the muscularis propria is observed. No cancer invasion into the periureteral adipose tissue (pT2). (B) D2-40 immunohistochemical staining (×200): Lymphatic invasion by urothelial carcinoma cells is evident (Ly1). (C) Hematoxylin and eosin staining (×20): Urothelial carcinoma involvement in a lymph node adjacent to the ureter (N1). (D) Hematoxylin and eosin staining (×40): No tumor cells are present at the ureteral margin (RM0).

Histopathological analysis revealed high-grade urothelial carcinoma with muscularis propria invasion (pT2), lymphovascular invasion (LVI1), negative ureteral margin (u-lt0), negative surgical margin (rm0), and one lymph node metastasis in the peritumoral adipose tissue surrounding the ureter (n1, N1) (Figure 5).

Adjuvant chemotherapy with gemcitabine and cisplatin was initiated but discontinued after one course due to adverse effects. The patient was followed postoperatively with contrast-enhanced CT imaging, routine blood work, and urine cytology every three months. Cystoscopy was performed as clinically indicated. At 24 months postoperatively, the patient remained free of recurrence, with stable renal function and no procedure-related complications.

## Discussion

Horseshoe kidney is one of the most common renal anomalies, occurring in approximately 0.2% of the population. Its characteristic anatomical features-including fusion of the lower poles and aberrant vascular courses-pose significant surgical challenges, particularly during procedures for upper tract urothelial carcinoma (UTUC) [4,5]. Furthermore, patients with horseshoe kidneys are reported to have a higher incidence of UTUC compared to the general population [6].

In the present case, although no major abnormalities were observed in the renal arteries and veins, one aberrant vessel was identified, originating from the distal portion of the right renal vein and extending toward the pelvis. Due to the fusion of the lower poles, horseshoe kidneys are often associated with multiple abnormal vessels, which may enter the kidney from various directions, resulting in a complex anatomical structure [7]. Therefore, accurate identification of vascular anatomy through detailed preoperative imaging, particularly CT angiography with 3D reconstruction, is essential for avoiding intraoperative vascular complications.

Moreover, the isthmus of a horseshoe kidney contains functioning renal parenchyma in approximately 80% of cases, as opposed to being merely a fibrous connection [7]. Consequently, when dividing the isthmus, careful resection and secure closure are critical to ensure adequate hemostasis and prevent urinary leakage. Robotic assistance facilitates this process through precise dissection and suturing under a magnified 3D view.

Historically, open surgery was the standard approach for treating UTUC in patients with horseshoe kidneys due to anatomical complexity. However, advancements in minimally invasive techniques, particularly robot-assisted surgery, have enabled safe and effective approaches in such complex cases, as demonstrated in both robot-assisted [4,5] and laparoscopic reports. Laparoscopic surgery, though less invasive than open surgery, can be limited by restricted visualization and instrument articulation, increasing the risk of bleeding and conversion to open surgery, especially in patients with complex renal anatomy [8].

In contrast, robot-assisted surgery offers several advantages: high-resolution three-dimensional imaging, tremor elimination, and articulated instruments that enable fine dissection and manipulation of structures with multiple degrees of freedom. These features are especially beneficial for managing abnormal vasculature and performing precise isthmus transection in horseshoe kidneys. Teo et al. reported that RANU improves the ability to manage anomalous vessels safely and contributes to reducing intraoperative bleeding and postoperative complications [3]. Additionally, multicenter studies have shown that robot-assisted approaches outperform traditional methods in terms of operative time, estimated blood loss, and postoperative recovery [4,9].

As the isthmus of a horseshoe kidney is often traversed by numerous vessels, preoperative CT angiography and 3D imaging are strongly recommended for surgical planning [7]. In this case, vascular assessment allowed safe identification and ligation of the aberrant vessel during robotic dissection. The procedure was completed without complications, demonstrating the utility of robotic technology in anatomically complex cases.

To date, only three previous case reports of RANU for UTUC in patients with horseshoe kidneys have been published in major databases [1,4,5]. Latif et al. first demonstrated the feasibility and safety of this approach, and Shiode et al. reported its usefulness in conjunction with a literature review. Our case represents the fourth such report and reinforces the growing body of evidence supporting the use of robotic surgery in this rare but challenging context. As more cases are reported, it will be possible to standardize the surgical technique and evaluate long-term oncologic outcomes.

In our case, robotic assistance allowed for the safe and precise management of both the aberrant vessel and the renal isthmus. Although systematic lymph node dissection was not performed, nodal metastasis was incidentally confirmed through the en bloc excision of surrounding tissues, including peritumoral adipose tissue. This outcome likely reflects the ability of robot-assisted surgery to achieve meticulous resection with adequate margins under a magnified three-dimensional field of view and enhanced instrument precision. According to the 2024 NCCN Guidelines, lymph node dissection is valuable for accurate staging even in clinically node-negative (cN0) patients; however, its benefit in improving survival remains uncertain. Therefore, a selective approach based on risk stratification is recommended [10]. According to a multicenter study by Margulis et al., lymphadenectomy during RNU improves the accuracy of pathological staging but does not consistently correlate with improved oncological outcomes. Therefore, a selective approach based on individual risk stratification is considered appropriate in cN0 cases [11]. Although adjuvant chemotherapy with gemcitabine and cisplatin was initiated, it was discontinued after one course due to adverse effects. Importantly, no recurrence has been observed during the 24-month postoperative follow-up, indicating successful local control of the disease. This outcome highlights the importance of thorough preoperative assessment, meticulous surgical execution, and effective perioperative management.

Robot-assisted RANU represents a promising surgical option for UTUC associated with complex renal anomalies such as horseshoe kidney, and its potential application may extend to other congenital anomalies, including ectopic kidneys and duplicated collecting systems. However, this report presents a single case, and outcomes may vary depending on tumor location, vascular anatomy, and the surgeon’s experience. Additionally, functional renal assessment was not quantitatively evaluated in this case. Further case accumulation through multi-center registries or pooled analyses may help establish standardized surgical protocols and clarify long-term outcomes.

## Conclusions

Robot-assisted nephroureterectomy is a safe and effective treatment option for UTUC in patients with horseshoe kidneys. Detailed preoperative vascular imaging, combined with the enhanced capabilities of robotic surgical systems, enables precise dissection and vascular control. This approach helps minimize intraoperative complications while supporting optimal oncological outcomes. As experience with robotic techniques continues to accumulate, these methods may become the preferred modality for treating UTUC in patients with complex renal anomalies.
